# An Innovative Possibilistic Fingerprint Quality Assessment (PFQA) Filter to Improve the Recognition Rate of a Level-2 AFIS

**DOI:** 10.3390/e25030529

**Published:** 2023-03-19

**Authors:** Houda Khmila, Imene Khanfir Kallel, Eloi Bossé, Basel Solaiman

**Affiliations:** 1 Control and Energy Management (CEM Lab), Sfax Engineering School, University of Sfax, BP W, Sfax 3038, Tunisia; 2 Smart Aid Technologies SATECH, Sfax 3061, Tunisia; 3 Image & Information Processing Department (iTi), IMT-Atlantique, Technopôle Brest Iroise CS 83818, CEDEX, 29238 Brest, France; 4 Expertises Parafuse Inc., Quebec, QC G1W 4N1, Canada

**Keywords:** fingerprint recognition, possibility distribution, minutia, level 2 features, AFIS, image quality assessment

## Abstract

In this paper, we propose an innovative approach to improve the performance of an Automatic Fingerprint Identification System (AFIS). The method is based on the design of a Possibilistic Fingerprint Quality Assessment (PFQA) filter where ground truths of fingerprint images of effective and ineffective quality are built by learning. The first approach, QS_I, is based on the AFIS decision for the image without considering its paired image to decide its effectiveness or ineffectiveness. The second approach, QS_PI, is based on the AFIS decision when considering the pair (effective image, ineffective image). The two ground truths (effective/ineffective) are used to design the PFQA filter. PFQA discards the images for which the AFIS does not generate a correct decision. The proposed intervention does not affect how the AFIS works but ensures a selection of the input images, recognizing the most suitable ones to reach the AFIS’s highest recognition rate (RR). The performance of PFQA is evaluated on two experimental databases using two conventional AFIS, and a comparison is made with four current fingerprint image quality assessment (IQA) methods. The results show that an AFIS using PFQA can improve its RR by roughly 10% over an AFIS not using an IQA method. However, compared to other fingerprint IQA methods using the same AFIS, the RR improvement is more modest, in a 5–6% range.

## 1. Introduction

**Figure 1:**
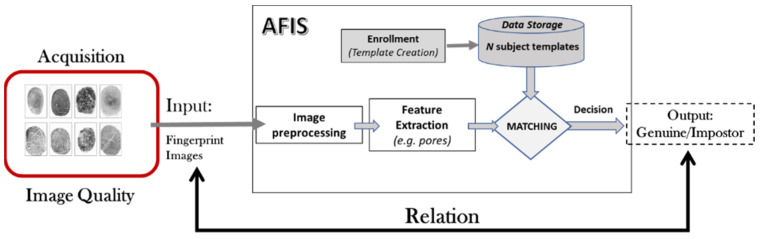
Generic block diagram of an AFIS.

Biometrics [1,2,3,4] is a technique for identifying people based on the measurement of their morphological, behavioral, or biological characteristics. There can be several characteristics, some of which are more reliable than others, but all must be unique to represent a single individual. The most sophisticated security and access control systems of our time are based on biometric systems. Fingerprint identification is one of the best-known biometric methods [5]. Fingerprints have been used to identify people for more than a century. Let us cite [6]: “*Fingerprint identification is based on two properties, namely, uniqueness and permanence as written in. It has been suggested that no two individuals (including identical twins) have the exact same fingerprints. It has also been claimed that the fingerprint of an individual does not change throughout the lifetime, with the exception of a significant injury to the finger that creates a permanent scar*”. Advances in computer capabilities have made fingerprint identification systems more automated; hence, they are named Automatic Fingerprint Identification Systems (AFIS). A generic block diagram of an AFIS is presented in Figure 1. An AFIS is generally composed of the following modules:

- *Acquisition*: a digital representation (images) obtained from a fingerprint scanner.
- *Feature extraction*: usually following a module to improve the image quality (preprocessing). A feature extractor further processes the raw digital images (samples) to generate a compact representation called a feature set to facilitate matching.
- *Enrollment (template creation)*: the enrollment module organizes one or more feature sets into an *enrollment template* that will be stored. The enrollment template is sometimes also referred to as a *reference*.
- *Data storage*: is devoted to storing templates and other demographic information about the user.
- *Matching*: this module takes a feature set and an enrollment template as inputs and computes the similarity between them in terms of a *matching score*. The matching score is then compared to a *threshold* to make the final decision; if the match score is higher than the threshold, the person is recognized (otherwise, the person is not).

The fingerprint image consists of black and white lines frequently referred to as ridges and valleys. The fingerprint’s characteristics are classified into three levels based on the shape of the ridges and their appearances. Level 1 features (pattern) are characterized by ridge flow shapes such as orientation field and singular points. Level 2 features (minutia points) are the terminations and bifurcations of ridges. Level 3 features (pores and ridge contours) are the finest details of the ridges [6]. These details can be obtained only with high-resolution sensors. Recently, fingerprint sensors have developed from an average resolution of 500 dpi (Dots per inch often confused with pixels per inch) to a high resolution of 1000 dpi and more. The high resolution of fingerprint images has made it possible to highlight level 3 features, such as pores, which are used to improve the performance of AFIS.

In general, an AFIS is based on the orientation and position of the Level 2 minutiae in the fingerprint image to make the match. The accuracy of this information is high, provided that the fingerprint image is of high quality. However, fingerprint images can be affected by degradation factors (injury, dirt, moisture, and dryness). Several researchers have been particularly interested in improving the quality of fingerprint images, usually through preprocessing and enhancement procedures of these data [7,8,9]. Thus, recent works propose image filtering to reject the input images whose quality does not meet a minimum quality requirement. Fingerprint image quality remains a significant challenge, even though several measurement processes have been proposed in the literature [10,11,12,13,14].

The abundance and diversity of sensors and techniques used during the acquisition can produce images of different qualities. However, the notion of quality in recognition systems remains strongly linked to the application. Indeed, an image can be helpful in one application but not in another. We can take the example where an iris image is used for a medical application, such as cataract recognition, which has a very low resolution, to identify individuals (biometric application). Indeed, in the context of a given application, the same image can lead a system to a correct decision and induce an error using another approach. Therefore, we cannot discuss an absolute quality (good/bad). The quality of an image is dependent on the application and the method used for processing. To estimate the quality of an image, one can intuitively refer to the objective of the recognition system that processes it.

**Figure 2:**
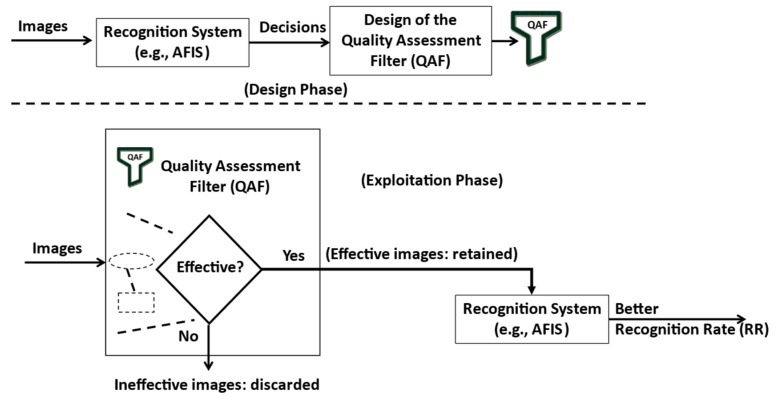
The general idea behind using an image quality assessment (IQA) filter.

In general, the objective of a recognition or classification system is to make correct decisions regarding the classification of images in predefined classes or not. Thus, if the decision taken is correct, it means that the system is performing well for the quality of the input image. On the other hand, if the decision taken is incorrect, it means that either the system admits a failure or that the quality of the input image needs to be better adapted to meet the system goal. Therefore, we propose in the present work to improve the performance of a recognition system (e.g., AFIS) without intruding on its processing to select or reject images based on their quality to fulfill the system’s goal. In other words, we propose to design a non-intruding image quality filter tailored to the decisions of a recognition system. The idea is illustrated in Figure 2.

Effectiveness is defined as ‘*the degree to which something is successful in producing a desired result or a success*’. In this work, the quality of an image is seen through that notion of effectiveness. In this work, we use the fingerprint biometric domain to demonstrate the value of our proposed quality assessment filter. For an AFIS, an image is considered effective if it leads to a correct identification decision and is ineffective when leading to an incorrect decision. Therefore, we expect to improve the AFIS recognition rate (RR) by discarding ineffective fingerprint images. We use 1st and 2nd order possibilistic modeling tools to assess and exploit the image quality, so we call our method Possibilistic Fingerprint Quality Assessment (PFQA).

The paper is organized as follows. Section 2 presents previous work related to fingerprint image quality assessment. Section 3 describes our proposed Possibilistic Fingerprint Quality Assessment (PFQA) filter. Section 4 shows the performance of our PFQA approach tested on experimental databases as well as a comparison with four fingerprint image quality assessment (IQA) methods. The conclusion is provided in Section 5.

## 2. Previous Work on Fingerprint Image Quality Assessment 

The performance of fingerprint-based recognition systems is strongly influenced by the condition of the fingertip surface, which varies according to environmental conditions or other causes. Below is a summary of some well-known and recent approaches to fingerprint quality assessment.

The NIST Fingerprint Image Quality (NFIQ) [15] is a widely used and publicly available metric for assessing image quality. It employs an Artificial Neural Network that takes 11 features extracted from the fingerprint image as input. These features are mostly related to minutia, and they are obtained from the output of the MINDTCT function of the NIST Biometric Image Software (NBIS). The NFIQ technique uses classification to categorize images into five classes, with a score of one indicating the highest quality and five the lowest. The primary objective of developing NFIQ was to assess biometric samples’ usefulness and predict the matching performance.

The NFIQ has been recently updated to NFIQ 2.0 [16], a collaborative effort between NIST and various government, public and private entities. NFIQ 2.0 also uses a classification-based approach, utilizing the OpenCV implementation of the Random Forest Classifier. In addition, it incorporates many features from previous fingerprint image quality methods to train the random forest classifier, using 14 features and match scores from the different commercial matching systems. The output values of NFIQ 2.0 range from 0 to 1, where a score of 1 indicates high utility and 0 denotes low utility for a given fingerprint image.

Chen et al. [17] propose a Local Clarity Score (LCS) that calculates the clarity of ridges and valleys per block by applying linear regression to determine a gray level threshold, classifying pixels as ridges or valleys. Next, a ratio of misclassified pixels is determined by comparing it to the normalized width of the ridges and valleys in that block. The work presented by Lim et al. [18] consists of computing the following features in each block: Orientation Certainty Level (OCL), ridge frequency, ridge thickness, and ridge thickness ratio to valley thickness. The blocks are labeled as good, indeterminate, poor, or empty by setting thresholds for the four features used. A Local Quality Score (LQS) is finally calculated from the total number of blocks classified as good, poor, and indeterminate quality for this image. However, the proposed quality metric also involves several thresholds to organize the local blocks into different levels.

Another approach was proposed by Chen [17], based on the orientation flow (OFL) in an image. The method relies on the observation that the direction of ridge flow changes gradually in high-quality fingerprint images. The technique involves calculating the orientation differences between a block and its neighboring blocks, referred to as the local Orientation Quality. The final quality score is obtained by averaging all the local Orientation Quality values.

Yao et al. [19] propose an approach based on the minutiae template only, in which the convex hull and Delaunay triangulation are adopted to measure the area of an informative region. This algorithm is, therefore, dependent on minutiae extraction operation. The authors of [20] offer another quality metric based on multiple segmentation. They performed a two-step operation on a fingerprint image, including segmentation and a pixel pruning operation. The pixel pruning is implemented by classifying the quality of the fingerprints into two general cases: the desired image and the undesired image. Teixeira and Leite [21] recently proposed a quality estimator for high-resolution images. Sharma et al. [22] propose to extract some features based on the distribution of ridges and valleys (moisture, uniformity of ridge and valley area, number of ridge lines, etc.). They use these features and a decision tree to detect different quality blocks (dry, good, normal dry, wet, and normal wet).

Andrezza et al. [23] propose an approach based on the Gabor filter analysis. Numerous convolution iterations are applied to the fingerprint, the filtered images are combined, and the homogeneity of the resulting image is calculated to determine the quality score. The disadvantage of this approach is the excessive computation time. Sharma et al. [24] suggest using the Local Phase Quantization (LPQ) descriptor to determine the quality score. Their work indicates that local descriptors are well-suited for evaluating the texture quality of fingerprint images. Finally, Panetta et al. [25] present a Local Quality Measure (LQM) based on fingerprint image features, which include sharpness, contrast, orientation certainty, symmetry characteristics, information about the symmetry, and information about the structure of friction ridges (minutiae). 

Lim et al. [26] use Frequency Domain Analysis (FDA) to evaluate the quality of fingerprint images. They discovered that high-quality images possess a single dominant frequency, while poor-quality images have a dominant frequency at low-frequency values or no single dominant frequency. A different Frequency Domain Analysis technique is presented in [27], known as the Radial Power Spectrum (RPS) method. This approach transforms the image into the frequency domain using a 2D Discrete Fourier Transform (DFT). The quality value is then determined by calculating the entropy of the energy distribution in the frequency domain, where a region of interest is defined as an annular band in a power spectrum.

Shen et al. [28] introduced a Gabor Feature-based Fingerprint image quality metric, known as the Gabor Shen (GSH) method. This technique involves computing m Gabor features for each block in an image. If all m Gabor features have similar responses, the block is considered to be of poor quality. On the other hand, if the m Gabor features produce varying responses, the block is regarded as being of good quality. The standard deviation of these Gabor feature blocks is used to differentiate each block into foreground and background blocks. The quality score is finally calculated as the ratio of the total number of good-quality blocks to the available foreground blocks. Olsen et al. [29] presented another Gabor feature-based method called the GAB approach. This method applies Gabor filters with four orientations to the entire fingerprint image instead of the individual image blocks. The quality score is then obtained by computing the average of the standard deviations of the four Gabor responses of the entire fingerprint image. 

The list above is far from being exhaustive. Several other works exist on fingerprint image quality assessment [5,30,31,32,33]. However, most existing works require the computation of several quality-related elements to assess the quality of the fingerprint texture.

## 3. Design of a Possibilistic Fingerprint Quality Assessment (PFQA) Filter

**Figure 3:**
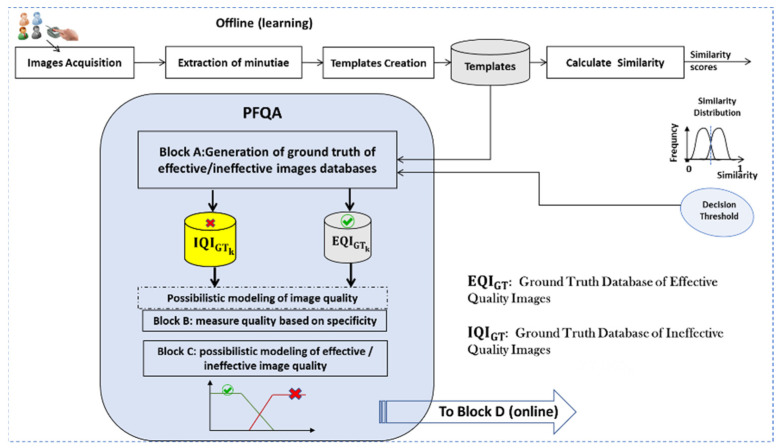
The design process of the PFQA filter (offline phase).

**Figure 4:**
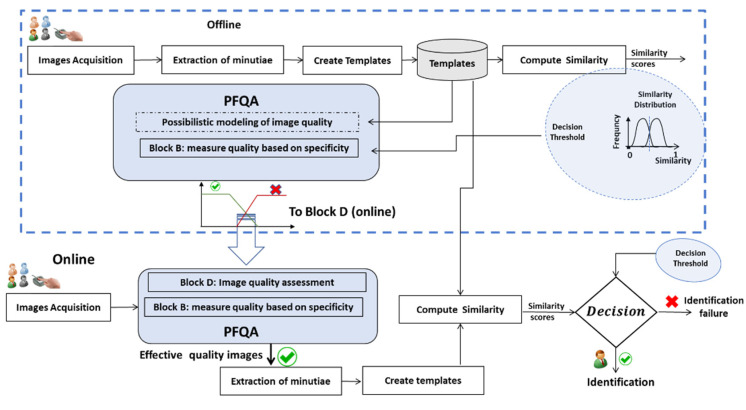
Using the PFQA filter (online) to improve AFIS decisions.

The goal of an AFIS is to identify people based on the texture of their fingerprints. A generic architecture of an AFIS has been presented in Figure 1. The fingerprint is still the most widely used biometric modality for identification. Therefore, developing new approaches to improve AFIS performance is a hot research topic. Our approach, based on PFQA, consists of designing a filter rejecting, which an AFIS does not give a correct identification decision. These images are therefore considered of ineffective quality to achieve the AFIS goal. Figure 3 presents the design process of the PFQA filter, which is applied during the learning phase (offline), while Figure 4 shows its online usage for improving the recognition rate (RR) of an AFIS.

The PFQA design requires four key subsystems identified as Blocks A, B, C, and D. These four blocks are detailed below.

Block A—Generation of the ground truth of effective/ineffective image databases. The ground truths of image quality are generated based on the AFIS decisions. The effective quality images are obtained from the AFIS decisions (true positive and true negative). Conversely, images of ineffective quality are derived from incorrect decisions (false positive and false negative) of the AFIS.

Block B—The fingerprint image texture quality measurement subsystem: the quality is measured by calculating the amount of uncertainty contained in the model of a contextual quality indicator (CQI) for a fingerprint image.

Block C—Subsystem to build quality models of attribute behaviors for effective/ineffective quality classes. Quality models are deduced from the models representing the individual behavior of the CQI within the fingerprint image.

Block D—Quality assessment subsystem. The quality of the fingerprint image is evaluated by projecting the quality measure deduced from the fingerprint image onto the constructed quality models.

### 3.1. Block A: Generation of Ground Truths for Both Effective and Ineffective Image Databases

As previously mentioned, in this work, the quality of an image is seen through that notion of effectiveness: *effective* if the image leads to a correct identification decision and *ineffective* if not. Consider the example of Table 1, which gives the matching result of an AFIS.

Matching the image M_1,1_ with M_2,1_ gives a correct decision. The same image M_1,1_, when matched with another image M_2,2_, gives an incorrect decision. We deduce that the image, M_1,1_ is effective with the matching (M_1,1_, M_2,1_) and ineffective for the matching (M_1,1_, M_2,2_). It illustrates our notion of effectiveness quality for an image. Our approach is to estimate an effective quality score (QS) for an image based on all the matching results performed by the AFIS. Thresholding the estimated scores for all the images allows discriminating between those having an effective quality to be well identified by the AFIS and those leading to incorrect identifications. This, in fact, results in the generation of two ground truth databases: effective images (templates) and ineffective images (templates), identified as $EQI_{GT}$ and $IQI_{GT},\;\mathrm{respectively}$, in Figure 3. 

The construction of $EQI_{GT}$ and $IQI_{GT}$ proceeds in two steps: (1) compute the quality scores of the effective images, and (2) establish a threshold on the quality scores for the effective images. Two techniques are used to compute the QSs. We propose two techniques. The first technique, called QS_I, is based on the AFIS decision for the individual image, and the second one considers the paired (or matched) image (QS_PI).

**Figure 5:**
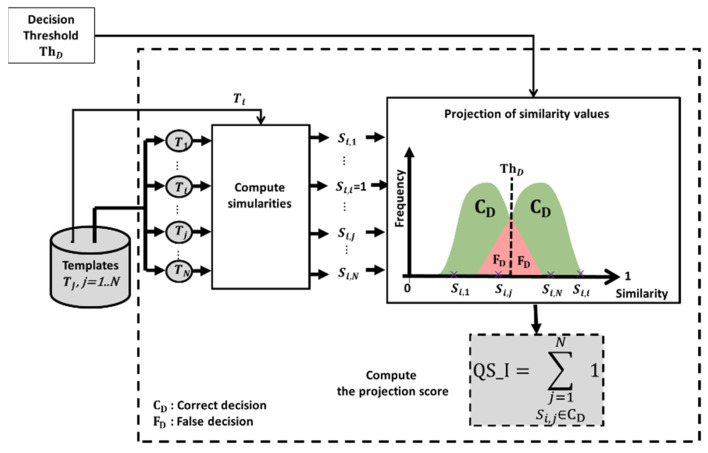
Computation of QS_I for the image $M_{i}$.

➢ Technique QS_I: According to the example in Table 1, each image can have correct and incorrect decisions depending on the matching. A QS of the image can be computed from the number of correct decisions. Figure 5 shows the process of calculating QS_I.

Figure 5 presents the steps of the QS_I computation process for an image *M_i_*. The template, $T_{i}$, of the image, *M_i_* is matched with all the other templates $T_{j},\;j=1\;\mathrm{to}\;N$, using a similarity measure. The quality score, QS_I, corresponds to the total number of all correct decisions that *M_i_* has induced, where *N* is the total number of images in the training database. $S_{i,j}$ is the similarity score between the image, *M_i_*, and an image, *M_j_*_._ The computed QS_I varies between $\left[QS\_I_{min},QS\_I_{max}\;\right]$ where $QS\_I_{min}=0$ and $QS\_I_{max}=N$. Higher is the value of $QS\_I\left(M_{i}\right)$, more effective is its image quality. If $QS\_I\left(M_{i}\right)=N,$ then *M_j_* is fully effective since it leads to all correct decisions. If $QS\_I\left(M_{i}\right)=0,$ then *M_j_* is fully ineffective since it leads to no correct decisions.

**Figure 6:**
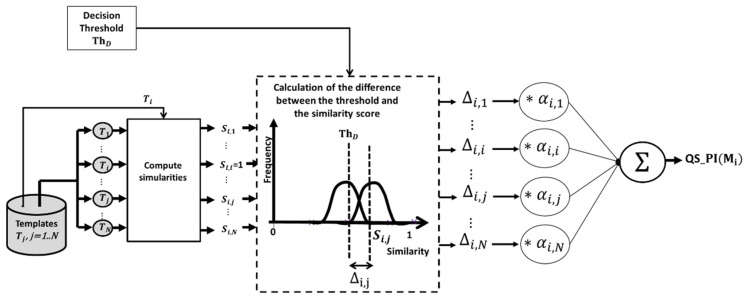
QS_PI calculation process for the image $M_{i}$.

➢ Technique QS_PI: An AFIS decision is made by matching a pair of images. Thus, the two images matched are equally responsible for the matching result. This aspect is considered in the calculation of QS_PI (Figure 6) by assigning a score to the pair, based on the deviation of their similarity value from the decision threshold, *Th_D_*.

Let the pair $\left\{M_{i},M_{j}\right\}$, having a similarity $S_{i,j}$, the difference $\Delta_{i,j}$ is determined as follows:

$$\Delta_{i,j}=\left\{\begin{array}{c} S_{i,j}-Th_{D}\;\;\;\;if\;\;\;\left\{M_{i},M_{j}\right\}\;\in same\;person\;\\ Th_{D}-S_{i,j}\;\;\;if\;\;\;\left\{M_{i},M_{j}\right\}\;\notin same\;person \end{array}\right.$$

The values of $\Delta_{i,j}$ vary between $\left[-1,1\right]$. The pairs of images with deviation values greater than 0 correspond to the correct decisions and those with values lower than 0 reach incorrect decisions. The larger $\Delta_{i,j}$, the more likely the image $M_{i}$ is to be of effective quality. The quality score QS_PI is a weighted average of the deviations $\Delta_{i,j}$.

(1) $$\;QS\_PI\left(M_{i}\right)=\overset{N}{\underset{j=1}{\sum }}\frac{\Delta_{i,j}*\alpha_{i,j}}{N}$$

$\alpha_{i,j}$, is a weight for the deviation $\Delta_{i,j}$, which depends on the number of times the image, $M_{i}$, has contributed t correct classifications $NC_{i}$ or false classifications $NF_{i}$. The weight $\alpha_{i,j}$, varies between $\left[\frac{1}{1+N},\;1+N\right]$ and impacts the calculation of the score. If the deviation $\Delta_{i,j}$ is important, it can be because of $M_{i}$ or because of $M_{j}$. So $\alpha_{i,j}$ is used to amplify the impact of $\Delta_{i,j}$ if it is due to $M_{i}$ or to weaken the impact of $\Delta_{i,j}$ if it is due to $M_{j}$. Figure 6 shows the different steps for calculating QS_PI using the following equations.

(2) $$\Delta_{i,j}=\left\{\begin{array}{c} \frac{1+NF_{i}}{1+NF_{j}}\;\;\;\;if\;\;\;\Delta_{i,j}<0\;\\ \frac{1+NC_{i}}{1+NC_{j}}\;\;\;if\;\;\;\Delta_{i,j}\geq 0 \end{array}\right.$$

(3) $$NC_{k}=\overset{N}{\underset{l=1}{\sum }}\left(1\;\;\;if\;\;\;\Delta_{k,l}\geq 0\;\;\right)$$

(4) $$NF_{k}=\overset{N}{\underset{l=1}{\sum }}\left(1\;\;\;if\;\;\;\Delta_{k,l}<0\;\;\right)$$

The calculated image quality scores are in the range: $\left[QS\_PI_{min},QS\_PI_{max}\right]$ with

(5) $$QS\_PI_{min}=\underset{\;}{min}\left[\left(Th_{D}-1\right),-Th_{D}\;\right]*\left(N+1\right)$$

(6) $$\;QS\_PI_{max}=\underset{\;}{max}\left[\left(1-Th_{D}\right),Th_{D}\;\right]*\left(N+1\right)\;$$

**Figure 7:**
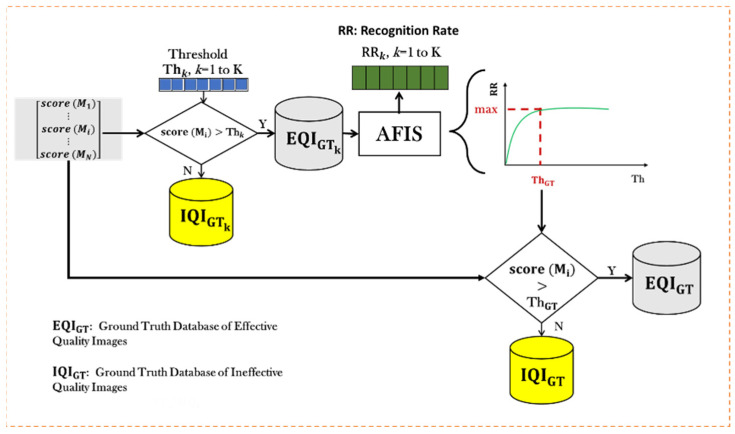
Determination of the threshold, $Th_{GT\;}$.

After computing the quality scores, QS_I (Figure 5) and QS_PI (Figure 6), of all images in the training database using algorithms one and two, it is necessary to determine a threshold, $Th_{GT\;}$, on the quality scores (QS) to classify the images into a ground truth database of effective quality images ($EQI_{GT}$) and a ground truth database of ineffective quality images ($IQI_{GT}$). Figure 7 shows the process used to optimize the choice of the threshold, $Th_{GT\;}$, to classify images (i.e., templates) into effective quality images and ineffective quality images with respect to the AFIS goal, which is a maximum Recognition Rate, RR.

The threshold, $Th_{GT\;}$, is selected in the interval $\left[QS_{min},QS_{max}\;\right]$. The selection is made by varying its value and by calculating each threshold value, $Th_{k\;}$, the recognition rate, $RR\left(Th_{k\;}\right)$ obtained by the AFIS on the effective quality images. The threshold, $Th_{GT\;}$, is the $Th_{k\;}$ that gives the maximum RR.

### 3.2. Block B: Measurement of the Fingerprint Texture Image Quality

**Figure 8:**
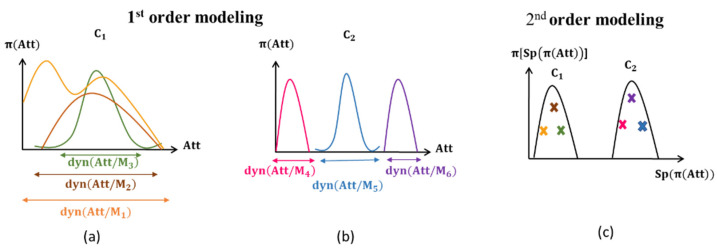
Possibilistic modeling of texture quality: (**a**,**b**) 1st order modeling and (**c**) 2nd order modeling.

Several approaches [17,18,19,21,22,23] have been developed for measuring fingerprint image quality. Most of them use texture attributes. In this paper, we propose a 2nd order possibilistic modeling to assess image quality. The 1st order modeling of the behavior of an attribute defines the spectrum of measures that the attribute can take on the texture for a class of quality. The 2nd order of possibilistic modeling gives another facet of the attribute behavior. Indeed, it allows specifying the variability of this spectrum (abbreviated as dyn in Figure 8), independently of its location within the definition domain of the attribute.

The models shown in Figure 8a,b show the first facet of the behavior of the attribute Att, represented by the possibility distribution of measurements taken on the image. The models given by (a) and (b) show no discrimination between the two quality classes C1 and C2 unlike the models given in Figure 8c. It is therefore clear that this facet of the behavior does not discriminate between C1 and C2. Indeed, the dispersion of Att measurements for the images belonging to the C1 class (M_1_, M_2_, M_3_), respectively, for the images belonging to the C2 class (M_4_, M_5_, M_6_) is not located at the same place for the Att domain values. However, it should be noted that the width of the distributions is related to the class: Att shows variations in the C1 class images and in the C2 class images, no matter what values it takes. However, some values are very close. This is the second facet of the Att behavior which is modeled as a second order (Figure 8c). 

Referring to the literature, one can easily notice the abundance of the use of global attributes generated from the gray level co-occurrence matrix (GLCM) in various works on the quality measurement of fingerprint images [20,34,35,36]. Therefore, we analyze the behavior of attributes within a texture and in response to stimuli. The sensitivity of the attributes is directly related to texture quality classes. Therefore, the attributes, which are sufficiently sensitive to the stimuli that can distinguish between the different quality classes considered, can be used as quality indicators for this kind of texture. These attributes are hereafter named Contextual Quality Indicators (CQI). Their use for this purpose is carried out in two ways, according to whether the analysis relates to global behavior or individual behavior.

The model of the individual behavior is a distribution of possibilities of the measurements that a CQI can take on different locations of the texture image. This model, therefore, represents the amount of uncertainty (attached to CQI) contained in the texture in question. Several measures have been proposed in the literature to quantify that uncertainty. Let us mention the specificity (Sp) [37], the uncertainty measure (U) [38], and the confidence index (Ind) [39]. In this paper, the measure of specificity, Sp, estimated for an image texture, is considered a measure of the quality of this texture. We call this quality measure SpQM.

Consider Ω to be the universe of discourse and $\pi \left(\Omega \right)$ to be the set of all possible distributions defined on Ω. The specificity measure, Sp, is a function Sp: $\pi \left(\Omega \right)\to \left[0,1\right]$, verifying the following properties:

$Sp\left(\pi \right)=1$, iff $\pi \left(x_{0}\right)=1$, and $\pi \left(x_{0}\right)=0\;\forall x\in \Omega \left(x\neq x_{0}\right)$;

$Sp\left(\pi_{\Phi }\right)=0$, iff $\pi_{\Phi }\left(x\right)=0$, ∀x∈ω;

If $\pi_{1}\leq \pi_{2}$, then $Sp\left(\pi_{1}\right)\leq Sp\left(\pi_{2}\right)$.

In light of this definition, several expressions of specificity have been proposed in the literature. One of the most used is given for a possibility distribution, π: $\Omega \to \left[0,1\right]$, such that $\pi \left(x_{1}\right)\geq \pi \left(x_{2}\right)\geq \cdots \geq \pi \left(x_{N}\right)$, normal and ordered, as follows: $Sp\left(\pi \right)=\pi \left(x_{1}\right)-\overset{K}{\underset{k=2}{\sum }}w_{k}\pi \left(x_{k}\right)$, where $\left\{w_{k}\right\},\;k=1,\;\cdots ,K$ is a set of weights with the following properties: $w_{k}\in \left[0,1\right]$, $\overset{K}{\underset{k=2}{\sum }}p_{k}=1;\;w_{k}\geq w_{t}\;\forall \left(1<k<t\right)$.

**Figure 9:**
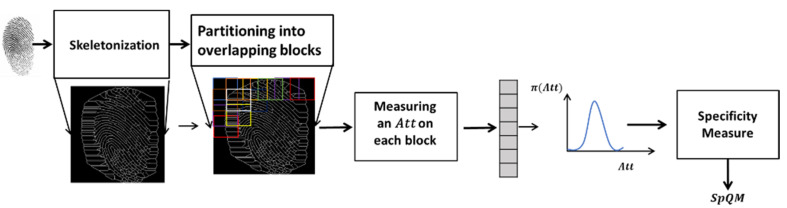
Steps to compute SpQM.

The quality measurement of the fingerprint texture is provided by the SpQM measurement. Figure 9 shows the main computational steps for this measure.

The fingerprint image is first skeletonized and then partitioned into overlapping blocks. The statistical attributes extracted from the GLCM matrix are used as contextual descriptors of the fingerprint texture. For each attribute Att, measurements are extracted from each block of the fingerprint image in order to build its individual behavior model within this image. The specificity measure of the model thus built represents the quality measure SpQM relative to the attribute Att.

### 3.3. Building Quality Models for Both Effective Quality Images (EQI) and Ineffective Quality Images (IQI)

**Figure 10:**
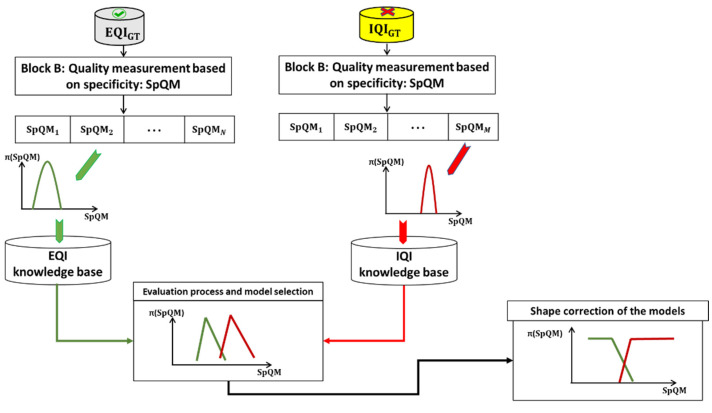
Construction of quality models.

The approach involves building two quality models, one for EQI and one for IQI, based on the ground truths ($EQI_{GT}$ and $IQI_{GT}$) and the quality measure SpQM. Figure 10 shows the construction process of the quality models of a fingerprint image relative to the two classes, EQI and IQI, for an attribute Att.

The construction of the knowledge bases relative to the effective/ineffective quality classes is carried out during the learning phase by considering all the attributes and by referring to the ground truth knowledge bases ($EQI_{GT}$: Effective, $IQI_{GT}$: Ineffective). At the end of this step, the system has as many models for each class as it has considered attributes. However, only one model for each of the two quality classes is required for the online phase. Hence, the use of an evaluation of each pair (effective/ineffective) relative to each attribute is considered. This evaluation allows the selection of the attribute to be considered as a CQI quality indicator for the AFIS.

The assumption on which the concept of quality is based in this paper is that the texture of the fingerprint is considered to be of effective quality if the AFIS can assign the correct identification decision. The texture is of ineffective quality if the AFIS fails to classify it. This hypothesis leads us to link the good partition of the two classes, EQI and IQI, to a maximization of the recognition rate of the AFIS computed on the EQI.

The selection process of a pair of models is thus designed to maximize the recognition rate of the AFIS on the EQIs. The process of selecting a pair of models is therefore designed to maximize the recognition rate of the AFIS on the EQIs while rejecting a reasonable number of images in IQI. The full process is detailed in Khmila’s thesis [40]. For each pair of models from an attribute Att, t = 1 to T, a projection of the quality measures of all the images of the learning database is performed in order to retain those considered of effective quality and to make them pass by the AFIS and to reject those considered of ineffective quality (a notion of filtering). A recognition rate and a rejection rate are thus generated for each pair of models. The representation of the recognition rates as a function of the rejection rates gives an attribute dispersion map (see details in [40]).

**Figure 11:**
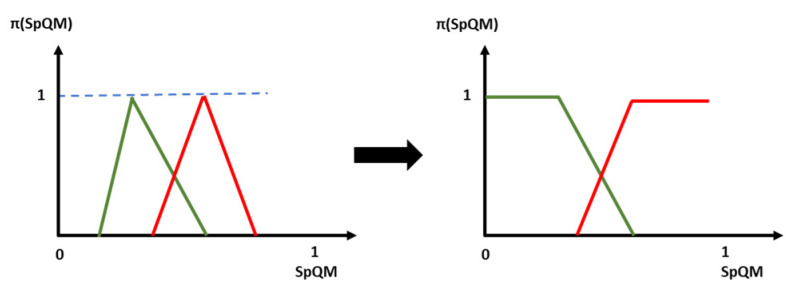
Quality model correction process.

The SpQM quality measures vary between [0,1]. In the case where the number of images in the training database is limited, the SpQM values do not cover all the values of the interval [0,1]. Indeed, few images would be available for the limit cases of 0 and 1. We then propose to correct the model shapes by completing them in the extreme zones, as in Figure 11. Figure 11 shows the process of correcting the quality models. This correction consists of assigning to the model that covers the high specificity area the maximum possibility (equal to 1) at any value of SpQM greater than the value of SpQM that corresponds to the maximum possibility of this model. -Assign, to the model covering the low specificity area, the maximum possibility (equal to 1) to any value of SpQM lower than the value of SpQM that corresponds to the maximum possibility of this model.

### 3.4. Quality Assessment (Block D)

**Figure 12:**
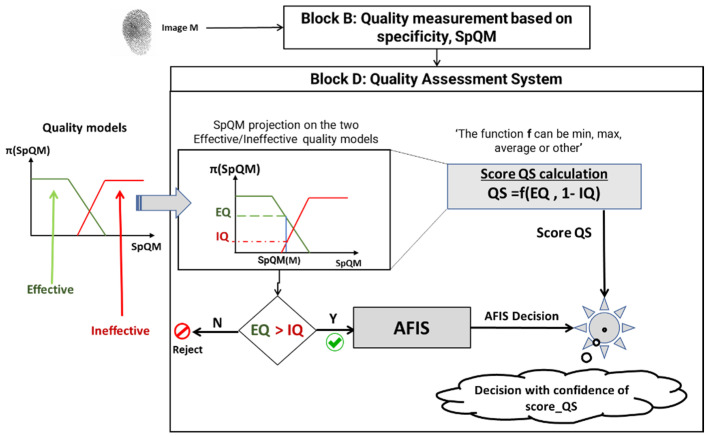
Quality assessment system.

When using the PFQA, the quality of each new image is evaluated, and its quality score is computed by the quality evaluation subsystem, as shown in Figure 12.

First, the quality of the input image M is measured by Block B. The resulting SpQM value becomes the input of the quality evaluation Block D. SpQM is then projected onto the quality models generated and selected in the learning phase. The possibility values EQ and IQ for this image to be of effective quality or ineffective quality are derived from this projection. If the image is evaluated as ineffective quality (EQ < IQ), it is rejected; otherwise, it passes through the AFIS and contributes to the identification process. In this case, a quality score of the image score_Q is calculated as a function of (EQ and IQ). This score is assigned to the AFIS decision as a weight quantifying the confidence to be given to this decision.

## 4. Experimental Results

### 4.1. Two Experimental Fingerprint Databases

The proposed PFQA approach is evaluated and tested on two fingerprint databases: CASIA-FingerprintV5 [41] and FVC2002DB1 [42]. The CASIA-FingerprintV5 database contains 20,000 fingerprint images of 500 individuals. The fingerprint images in CASIA-FingerprintV5 were captured in a single session using the fingerprint sensor URU4000. CASIA-FingerprintV5 volunteers are graduate students, workers, servers, etc. Each volunteer provided 40 fingerprint images of their eight fingers (left and right, thumb/second/third/fourth finger), i.e., five images per finger. The volunteers were asked to rotate their fingers with different levels of pressure in order to generate significant intra-class variations. All fingerprint images are BMP files of 8 bits of gray level and size 328 × 356, with a resolution of 500 dpi.

In our study, we used 100 individuals. For each individual, we take five images of the right thumb. We obtained a database of 500 images, which is largely sufficient to test our approach. This database is partitioned into a learning database and a test database. For the learning database, we take for each individual the first three images (a total of 300 images), and for the test database, we take the two remaining images for each individual to obtain a total of 200 images.

The FVC2002DB1 fingerprint database [42] contains 80 fingerprint images of 388 × 374 pixels, with a resolution of 500 dpi. The images were acquired from 10 users (8 acquisitions for the same finger user), via the Identix TouchView II scanner. To further prove the effectiveness of our approach, we have degraded the quality of the images of the FVC2002DB1 database by adding a circular blur noise. Since the number of images in this database is limited, we duplicated them by making translations and rotations in order to increase the number of images. As a result, we obtained for each individual 16 images instead of 8 images. We name this database FVC Degraded (FVCD). This database is also partitioned into a training database and a test database, each containing eight images for each individual.

### 4.2. Experimental Setup with Two Conventional AFIS: AFIS1 and AFIS2 

Our experiments use two existing AFIS: AFIS1 [43] and AFIS2 [44]. They are two “open source” systems. The first system is basic. It first involves aligning the fingerprints and then matching the minutiae. The second system uses contextual information provided by the ridge stream and the orientation in the neighborhood of the minutiae detected in the fingerprint image to compute the score between the two images.

Our PFQA approach uses the AFIS recognition rates (RR) to determine the parameters involved in the modeling and decision-making steps. However, in an experimental setting, unlike the real-world setting, we have databases of a limited number of images. The number of images in the database then presents a constraint to consider because a relatively large rejection rate can lead to an evaluation of the AFIS on very few images, making the notion of recognition rate relatively insignificant. Therefore, in the experimental framework in which we carry out the present experiments, we choose to make a compromise between a recognition rate (RR) of the AFIS that we want to maximize and the image rejection rate (IRR) that we wish to optimize in order to keep a significant number of images. The compromise consists of an equal rate between the recognition rate and the rate of images kept (effective quality images). We call it Equal Rate Compromise (ERC). 

### 4.3. Generation of Ground Truth Images 

The generation of the ground truths is carried out in two steps. The first step is calculating the quality score QS according to the following two methods: QS_I (Figure 5) and QS_PI (Figure 6). The second step consists of defining a threshold for the scores for each method.

#### 4.3.1. Selection of Thresholds on the Scores

**Figure 13:**
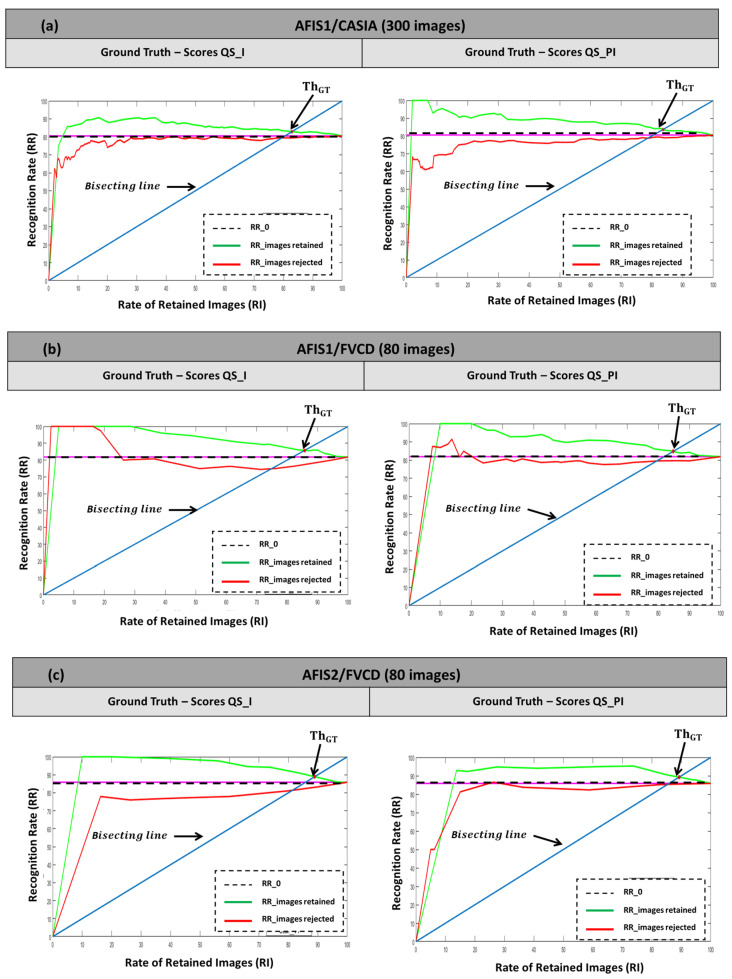
Performance of the PFQA approach: (**a**) AFIS1/CASIA, (**b**) AFIS1/FVCD, (**c**) AFIS2/ FVCD.

To choose a threshold $\left(Th_{GT}\right)$ on QS_I and QS_PI while considering the ERC, we represent the recognition rates of the retained images (RR_RI) as a function of the rates of the retained images (R_RI). The tests are performed on the two databases, CASIA and FVCD, and for the two approaches, QS_I and QS_PI. Threshold $\left(Th_{GT}\right)$ to be retained is the one for which RR_RI = R_RI. It is, therefore, the intersection of the curve with the first bisector. Note that the recognition rate of the AFIS without quality filtering is the value RR_0, which corresponds to R_RI = 100%. By observing the curves, we notice that for the six cases (AFIS/databases) presented in Figure 13, the filtering performed allowed a tangible improvement in the recognition rate of the AFIS. 

The RR improvement of the AFIS can be seen in the difference observed between the R_RI = f(R_RI) curve and the value RR_0. This difference is important for low R_RI rates. For example, a difference between 20% and 40% can be observed for the case illustrated in Figure 13a. It can reach up to 20% improvement (case of Figure 13b). In a real-world situation, one should select the threshold, $Th_{GT}$, as the value that allows having the maximum deviation, independently of the images rejected. However, in this experimental setup, subject to ERC, the selected $Th_{GT}$. Although it allows for retaining effective quality images, it does not correspond to our approach’s best or real performance.

In order to realize the impact that the rejected images have on the AFIS performance before discarding them, we show the AFIS RRs on these images. These are the recognition rates of the discarded images (red curves in Figure 13 as a function of (1—RR_RI)). The corresponding curves show an increasing trend to reach RR_0 at the point 0% of discarded images (R_RI = 100%). They present a difference with the initial value RR_0, which is becoming more important as there are more images judged to be of ineffective quality. This confirms that the filtered images were indeed of ineffective quality and are responsible for decreasing the AFIS performance.

#### 4.3.2. Construction of Quality Models Based on Ground Truths: ($EQI_{GT}$: Effective, $IQI_{GT}$: Ineffective)

**Figure 14:**
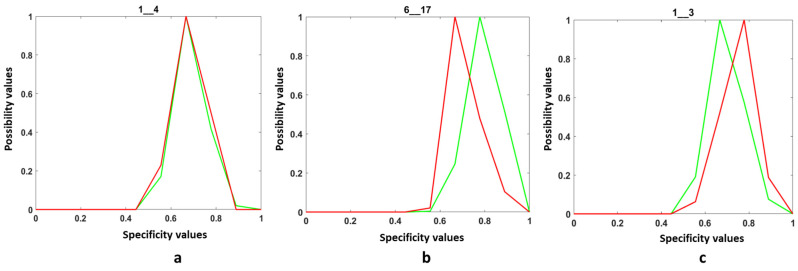
The three categories of quality models: (**a**) the two models are confused, (**b**) the EQI model is in the area of the highest specificity, and (**c**) the IQI model is in the area of the highest specificity.

The steps for constructing the quality effective/ineffective models are detailed in [40]. The quality models obtained are classified into three categories. The first category is the confused models (Figure 14a). In this case, there is no separation between effective and ineffective images. Discrimination is impossible. The second category is where the attribute specificity values in the effective quality images are higher than (Figure 14b) those of ineffective quality images, and finally, the third category is the one where the attribute specificity values computed for the ineffective quality images are higher than those of the attributes extracted from the effective quality images (Figure 14c). Figure 14 shows examples of three different attributes.

It is obvious that the models of the first category are useless. It means that the behavior of the corresponding attributes does not depend on the texture quality. The models of the second and third categories show that the corresponding attributes do not behave in the same way with effective quality images as with ineffective quality images, according to the AFIS goal. The patterns in the second category correspond to attributes that keep relatively stable values in effective quality textures and relatively fluctuating values in ineffective quality. This behavior is manifested in the specificity values of the distributions of these attributes on the image, which are more important for images of effective quality images. The opposite behavior characterizes the attributes according to the models of the third category.

#### 4.3.3. Evaluation Process of the Representative Models of the Quality Classes and Selection of the CQIs

The process of evaluating the models representing the ground truth images of the two classes’ effective/ineffective quality is described in Chap.3 of [40]. For reasons of space, we present here only the main lines. Attributes with models that are not discriminating models for the classes are discarded. The rate of discarded models is the ratio between the number of discarded models and the total number of attributes (16 × 19). Each retained attribute is represented by its coordinates (rejection rate, recognition rate) on the attribute dispersion map as described in Chap.3 of [40]. We perform the selection of the attributes related to the selected models, as shown in Table 2. The selection is made for the three (AFIS/database) pairs by the QS_I and QS_PI techniques, also considering the ERC trade-off. 

**Figure 15:**
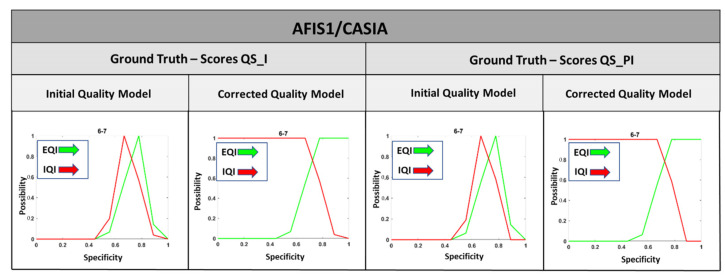
Example of shape corrections applied to the quality models.

The shape of the models adopted for the evaluation of the quality of the fingerprint images of each AFIS/database pair for both QS_I and QS_PI approaches is corrected to have quality models capable of covering all possible quality measures that an image may have. Figure 15 shows an example of shape correction applied to the quality models adopted by the QS_I and QS_PI approaches for the first row of Table 2 above. 

### 4.4. Performance Evaluation of the PFQA Approach

**Figure 16:**
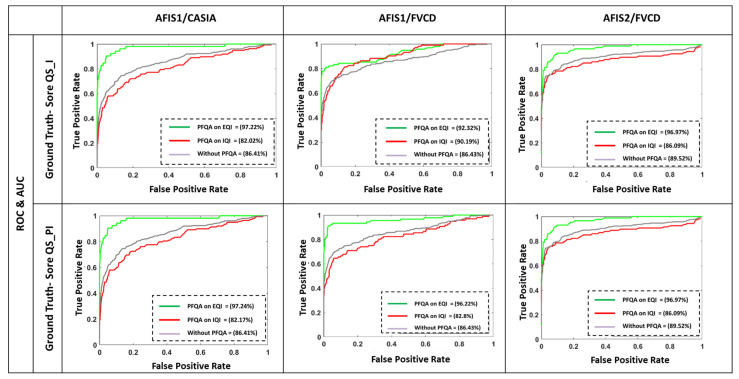
Comparison of ROC curves with and without PFQA.

**Figure 17:**
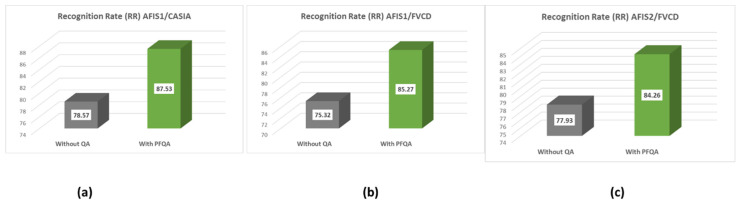
Histogram of AFIS recognition rates (RR) with and without the use of PFQA: (**a**) AFIS1/CASIA, (**b**) AFIS1/FVC2002, (**c**) AFIS2/FVC2002.

The PFQA filter is characterized by two CQI behavior models represented by two quality classes, EQI and IQI (obtained during an offline phase). This filter is used online at the AFIS front (as in Figure 4). In this experiment, we compare an AFIS performance with and without PFQA. Figure 16 shows the results of the similarity score distributions of the two classes True Positive (TP) and False Positive (FP) without PFQA (Figure 16—blue curves) and with PFQA (green curves—PFQA on effective images; red curves—PFQA on ineffective images). Figure 16 presents the performance of PFQA for the three combinations (AFIS/Database) for the two methods used to calculate the quality scores: QS_I and QS_PI. In all cases, the green curves where PFQA has been used to identify effective images show better results. When compared to the blue curves, the red curves show a negative impact on performance when processing ineffective images. Finally, the histogram in Figure 17 confirms the significant increase in AFIS performance when using PFQA.

### 4.5. Performance Comparison with Four Current FQA Approaches

**Figure 18:**
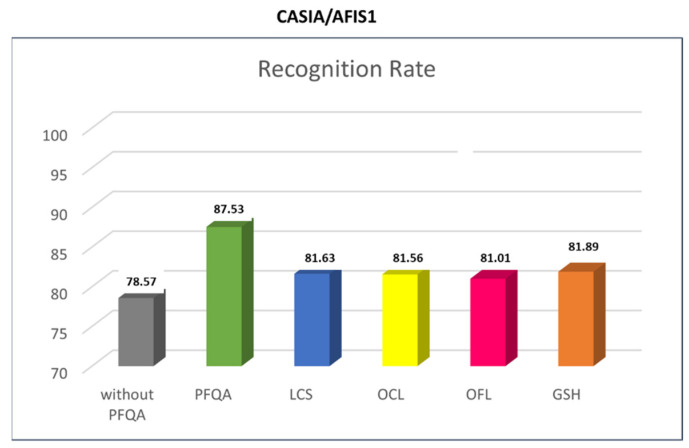
Histogram of AFIS1/CASIA recognition rates (RR) as compared to four current methods: LCS, OCL, OFL, and GSH.

The comparison is conducted on the AFIS1/CASIA pair and the PFQA filter designed with QS_PI. The CASIA database is chosen because it has a relatively large number of images. The PFQA filter is compared to four quality assessment methods: OCL (Orientation Certainty Level) [18], LCS (Local Clarity Score) [17], OFL (Orientation Flow) [17], and GSH (Gabor Shen) [28]. The histogram in Figure 18 shows the RR results of an AFIS1 using the four quality assessment approaches. All approaches show a RR improvement compared to the method without PFQA. PFQA outperforms all other approaches with a RR of 87.53%, so almost 10% compared to the method not using IQA (78.57%) and roughly 5–6% RR improvement compared to methods using IQA. 

**Figure 19:**
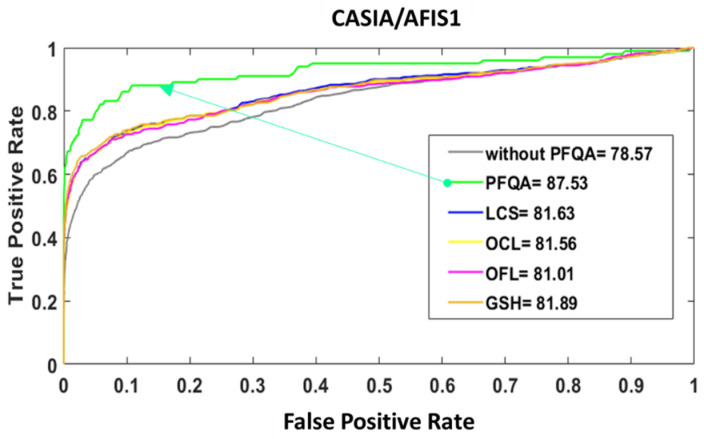
Comparison of ROC curves without/with PFQA and four current approaches for filtering bad-quality images for CASIA/AFIS1 pair.

The ROC curves and the areas under the ROC curves presented in Figure 19 confirm that the performance of PFQA is superior to the performance of the other approaches (LCS, OCL, OFL, GSH) considered in this paper for filtering the bad-quality images of the CASIA database.

## 5. Conclusions and Future Work

In this paper, we have proposed an innovative approach to improving the performance of an Automatic Fingerprint Identification System (AFIS). The method is based on the design of a Possibilistic Fingerprint Quality Assessment (PFQA) filter where ground truths of fingerprint images of effective and ineffective quality are built by learning. The first QA approach (QS_I) is based on the AFIS decision for an image without considering its paired image to decide its effectiveness. The second QA approach, QS_PI, is based on the AFIS decision but also considers its pair. These two ground truths (effective/ineffective) are used to design the PFQA filter. PFQA discards the images for which the AFIS does not generate a correct decision. The proposed filtering approach has been evaluated on two experimental databases using two conventional AFIS. In addition, a comparison of four known fingerprint IQA methods has been performed. The results show that an AFIS using PFQA can improve its recognition rate by roughly 10%. Compared to other methods using IQA, the improvement is more in the order of 5–6%. 

Future work will extend the performance evaluation to other domains than fingerprint, for instance, to multimodal biometric systems. Additional research is necessary to evaluate the effectiveness of these techniques, not only for the fingerprint characteristic but also for other biometric traits such as iris, face, palm, and others, for instance, to determine the appropriate image quality indicators that can effectively represent prior domain knowledge. It is also essential to assess how these indicators perform with various image textures and determine which CQIs are most relevant for specific domains or traits.

## Figures and Tables

**Table 1:** The matching result of the AFIS: (G: Genuine, I: Impostor, C_D_: correct decision, X: false identification).

		ID_1_				ID_2_			
		M_1,1_		M_1,2_		M_2,1_		M_2,2_	
ID_1_	M_1,1_	-		G	C_D_	I	C_D_	G	X
	M_1,2_	G	C_D_	-		I	C_D_	I	C_D_
ID_2_	M_2,1_	I	C_D_	I	C_D_	-		G	C_D_
	M_2,2_	G	X	I	C_D_	G	C_D_	-	

**Table 2:** Results of the selection of the most important attributes.

Pair (AFIS/Database)	QS_I (Selected Attribute)	QS_PI (Selected Attribute)
AFIS1/CASIA	6_7	6_7
AFIS1/FVC2002DB1	9_1	10_3
AFIS2/FVC2002DB1	2_5	2_5

## Data Availability

Not applicable.
